# Exogenous 1′,4′-*trans*-Diol-ABA Induces Stress Tolerance by Affecting the Level of Gene Expression in Tobacco (*Nicotiana tabacum* L.)

**DOI:** 10.3390/ijms22052555

**Published:** 2021-03-04

**Authors:** Teng Liu, Cai-Xia Li, Juan Zhong, Dan Shu, Di Luo, Zhe-Min Li, Jin-Yan Zhou, Jie Yang, Hong Tan, Xin-Rong Ma

**Affiliations:** 1 CAS Key Laboratory of Environmental and Applied Microbiology, Environmental Microbiology Key Laboratory of Sichuan Province, Innovation Academy for Seed Design, Chengdu Institute of Biology, Chinese Academy of Sciences, Chengdu 610041, China; liuteng0120@gmail.com (T.L.); licx@cib.ac.cn (C.-X.L.); zhongjuan@cib.ac.cn (J.Z.); shudan@cib.ac.cn (D.S.); luodi@cib.ac.cn (D.L.); lizm1@cib.ac.cn (Z.-M.L.); zhoujy@cib.ac.cn (J.-Y.Z.); yangjie@cib.ac.cn (J.Y.); 2 College of Life Sciences, Sichuan University, Chengdu 610041, China; 3 University of Chinese Academy of sciences, Beijing 100049, China

**Keywords:** 1′,4′-*trans*-diol-ABA, foliar spraying, drought stress, transcriptome, ABA signaling pathway, tobacco

## Abstract

1′,4′-*trans*-diol-ABA is a key precursor of the biosynthesis of abscisic acid (ABA) biosynthesis in fungi. We successfully obtained the pure compound from a mutant of *Botrytis cinerea* and explored its function and possible mechanism on plants by spraying 2 mg/L 1′,4′-*trans*-diol-ABA on tobacco leaves. Our results showed that this compound enhanced the drought tolerance of tobacco seedlings. A comparative transcriptome analysis showed that a large number of genes responded to the compound, exhibiting 1523 genes that were differentially expressed at 12 h, which increased to 1993 at 24 h and 3074 at 48 h, respectively. The enrichment analysis demonstrated that the differentially expressed genes (DEGs) were primarily enriched in pathways related to hormones and resistance. The DEGs of transcription factors were generally up-regulated and included the bHLH, bZIP, ERF, MYB, NAC, WRKY and HSF families. Moreover, the levels of expression of PYL/PYR, PP2C, SnRK2, and ABF at the ABA signaling pathway responded positively to exogenous 1′,4′-*trans*-diol-ABA. Among them, seven ABF transcripts that were detected were significantly up-regulated. In addition, the genes involved in salicylic acid, ethylene and jasmonic acid pathways, reactive oxygen species scavenging system, and other resistance related genes were primarily induced by 1′,4′-*trans*-diol-ABA. These findings indicated that treatment with 1′,4′-*trans*-diol-ABA could improve tolerance to plant abiotic stress and potential biotic resistance by regulating gene expression, similar to the effects of exogenous ABA.

## 1. Introduction

Phytohormones and their analogs are widely used in the production of crops and other plants, and their exogenous application has important effects on the promotion of growth and development, improvement of stress tolerance and increase in disease resistance [1]. Abscisic acid (ABA) is involved in diverse plant processes, including seed dormancy, plant growth, and fruit ripening among others [2,3]. ABA also plays a key regulatory role in the response of plants to various biotic and abiotic stresses [4,5], and it is known to serve as a stress hormone [6].

**Figure 1:**
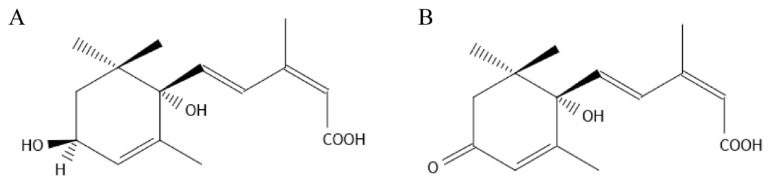
The chemical structures of 1′,4′-*trans*-diol-ABA (**A**) and ABA (**B**).

The chemical formula of 1′,4′-*trans*-diol-ABA is C_15_H_22_O_4_. There is only one hydroxyl difference between this compound and ABA (Figure 1). Walton obtained 1′,4′-*trans*-diol-ABA by chemical synthesis in 1972 [7]. Subsequently, 1′,4′-*trans*-diol-ABA was identified as an important precursor of ABA synthesis in many fungi. In *Botrytis cinerea*, it has been confirmed that 1′,4′-*trans*-diol-ABA was the final precursor of ABA [8,9]. In other fungi, such as *Cercospora rosicola* and *C. pini*-*densiflorae*, 1′,4′-*trans*-diol-ABA was also found and confirmed to serve as the biosynthetic precursor to ABA [10,11,12]. However, 1′,4′-*trans*-diol-ABA has not been found to be related to the known ABA biosynthetic pathway in plants, and there are only a few studies that confirmed these two compounds could be mutually transformed in different plants [13,14,15]. Owing to the similarity of its structural with ABA, it was hypothesized that 1′,4′-*trans*-diol-ABA had functions similar to those of ABA in plant growth regulation by finding its inhibitory effects on the growth of the excised embryonic axes of germinated bean [7] and rice seeds [16]. However, since it is difficult to obtain pure 1′,4′-*trans*-diol-ABA, the possible biological activity of this compound on plants remains unrevealed. In 2006, pure 1′,4′-*trans*-diol-ABA was isolated from a mutant strain of *B. cinerea*, and its crystal structure was confirmed by X-ray diffraction analysis [17]. Furthermore, through genetic improvement, we obtained an enhanced strain of *B. cinerea*, which could stably produce 1′,4′-*trans*-diol-ABA. We gradually increased its yield.

The adaptability of plant stress is primarily achieved by the regulation of gene expression. Therefore, one of the main methods to study whether a new compound can induce plant stress resistance is to explore its effects on the expression of related genes. ABA-mediated stress response is the main way in which plants tolerate stress. Plant have developed a complex gene regulatory network for the signal transduction of ABA. First, the stress-induced 9-*cis*-epoxycarotenoid dioxygenase (NCED) catalyzes the final rate limiting step that enhances the biosynthesis of ABA [18]. The response to ABA is initiated by a signaling pathway, which activates kinase cascades. ABA binds to a family of receptor proteins (pyrabactin resistance1 (PYR1)/PYR1-like (PYL)/regulatory components of ABA receptors (RCAR)) and promotes the formation of a complex of PYR/PYL/RCAR proteins and phosphatase 2Cs (PP2Cs). In the absence of ABA, sucrose non-fermenting 1-related protein kinase 2(SnRK2) protein kinases remain dephosphorylated; whereas, in the presence of ABA, ABA-bound receptors sequester PP2Cs, allowing the phosphorylation of SnRK2 and subsequent activation of ABA-responsive element binding proteins/factors (AREBs/ABFs) by phosphorylation [19]. The promoter of ABA-responsive genes possesses ABA-responsive elements (ABRE), which can bind to ABRE-binding proteins (ABREBPs) and activate transcription under ABA stimulation [20]. In addition, several transcription factors (TFs) of the bZIP, MYB, MYC, NAC, ERF, WRKY, and DREB protein families regulate gene expression in an ABA-dependent manner [21,22,23,24,25]. Typically, ABA-induced genes primarily encode the proteins associated with dehydrins and the enzymes that detoxify reactive oxygen species, enzymes of compatible solute metabolism, a variety of transporters, protein kinases and phosphatases, and the enzymes involved in phospholipid signaling. ABA suppressor genes encode proteins associated with growth, including cell wall, ribosomal, plasma membrane, and chloroplast proteins [20]. Whether 1′,4′-*trans*-diol-ABA also induces the expression of these ABA-related genes or other potential genes, thereby regulating plant tolerance to adversity stress, remains unclear.

To investigate the effect of exogenous 1′,4′-*trans*-diol-ABA on the expression of stress resistance genes in plants is of substantial significance for the study of the induction and mechanisms of stress tolerance.

Tobacco (*Nicotiana tabacum* L.) is an important economic crop and model plant. In this study, we tested the effect of 1′,4′-*trans*-diol-ABA on the enhancement of stress tolerance in tobacco and revealed its regulation on gene expression through a comparative transcriptome analysis. This study will provide a basis for further research on 1′,4′-*trans*-diol-ABA and its mechanism of inducing plant stress.

## 2. Results

### 2.1. Blade Stomatal Movement and Drought Plant Morphology

**Figure 2:**
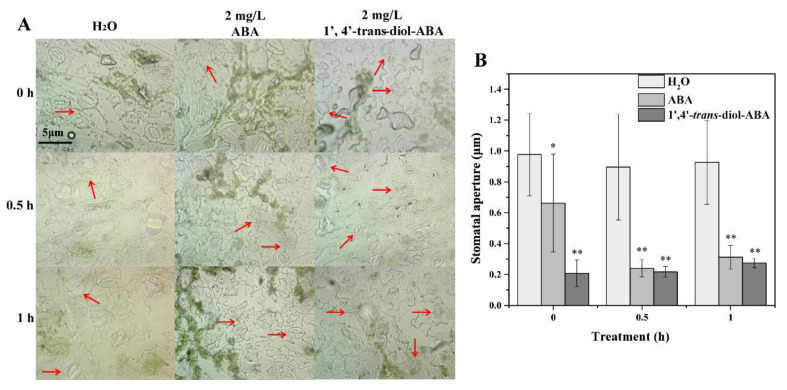
The effect on the stomatal opening of tobacco leaves by soaking with 1′,4′-*trans*-diol-ABA. (**A**) The stomata forms of the leaves in lower epidermis under the microscope observed. The magnification is 400×. Scale bar, 5 μm. The red arrows point stomata: In control group, most stomata are open; In treatment groups, most are closed. (**B**) Stomatal opening states under different treatments. The aperture 40 stomata per experiment are measured and counted. Asterisks (*) indicate significant difference from the control (H_2_O) at * *p* < 0.01 or ** *p* < 0.001.

Microscope observation revealed that the treatment of ABA and 1′,4′-*trans*-diol-ABA caused most stomata to close (Figure 2A). The measurement and statistics of stomata aperture show that compared with the control, 1′,4′-*trans*-diol-ABA significantly reduced the stomata opening, which was similar to the effect of ABA (Figure 2B).

**Figure 3:**
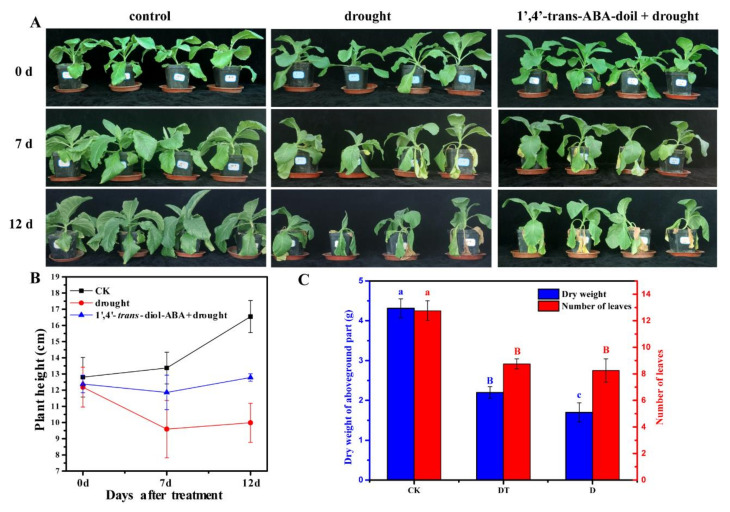
The effect of foliar spraying with 1′,4′-*trans*-diol-ABA on the drought tolerance of tobacco seedlings. (**A**) Morphological changes of plants under drought stress. The pictures from left to right were the control with normal watering, drought treatment, and co-treatment of 1′,4′-*trans*-diol-ABA spray and drought, observing the plant morphology at 0 d, 7 d and 12 d, respectively. (**B**) Plant heights at three time points exposed to different conditions. (**C**) The dry weights of aboveground part and number of leaves of the plants at 12 d after treatment. CK, control of keeping watering; DT, 1′,4′-*trans*-diol-ABA spray and drought treated; D, drought treated only. Different letters indicate significant differences among different treatments (lowercase, *p* < 0.05; uppercase, *p* < 0.01).

To understand the effect of 1′,4′-*trans*-diol-ABA on the drought tolerance of tobacco, a concentration of 2 mg/L (7.52 μmol/L) was used to spray the foliage. We found that drought suppressed the growth of plants, and the leaves wilted to varying degrees. However, 1′,4′-*trans*-diol-ABA significantly relieved stress symptoms (Figure 3A). When exposed to drought stress, the plant height and the dry matter quality of the aboveground parts were less than the control. However, compared with the drought group alone, the treatment of 1′,4′-*trans*-diol-ABA mitigated the damages and showed higher plant height and dry matter (Figure 3B). Moreover, the compound also made the plant retain a relatively large number of leaves (Figure 3C). This result preliminarily confirmed that 1′,4′-*trans*-diol-ABA could improve the drought tolerance of tobacco and alleviate the damage of drought to seedlings.

### 2.2. Data Analysis of RNA-Seq

**Figure 4:**
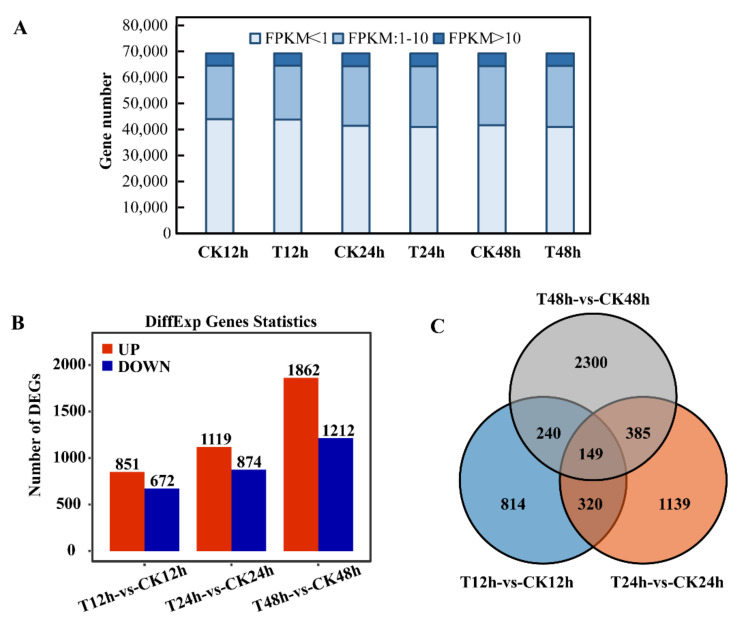
RNA-Seq statistics of 1′,4′-*trans*-diol-ABA and control leaves during three time points (12h, 24h, and 48h) after treatment. (**A**) Stacked bar chart of the number of transcripts in different FPKM value ranges in the sample; Bar plot (**B**) and Venn diagram (**C**) Illustrating the number of DEGs compared with the control at 12 h, 24 h, and 48 h after treatment. The statistical standard of DEGs was the FDR < 0.05, log_2_ (fold change) > 0.5 was up and log_2_ (fold change) < −0.5 was down. (T: 1′,4′-*trans*-diol-ABA treatment, CK: control).

Total RNA of the leaf samples was isolated, and all RNA integrity numbers (RIN) value were higher than 7.5. Eighteen cDNA libraries representing the control groups (CK12h, CK24h, and CK48h), 1′,4′-*trans*-diol-ABA treatment groups (T12h, T24h, and T48h) and their repetitions were constructed. Each of these libraries produced 39.0–49.65 million (M) raw reads. After filtering out the low-quality sequences clean reads of each sample were mapped to the tobacco genome. The unique mapping ratio ranged from 90.64% to 97.31%. Approximately 95% of the reads were observed in known exons and 5% were in predicted intergenic or intron regions. Detailed information on RNA sequencing and mapping is summarized in Table S1. A total of 69,213 transcripts were detected across all the samples. Approximately 31,000 transcripts with the fragments per kb per million reads (FPKM) ≥1 in at least one sample were collected for the following analyses (Figure 4A).

### 2.3. Analysis of Differential Expression Genes

Differential expression genes (DEGs) were identified from a comparison group between 1′,4′-*trans*-diol-ABA treated and the control group leaves at each time point (Figure 3B, Table S2). A total of 5348 non-redundant DEGs were obtained by pairwise comparison group, including 1523 DEGs in the 12 h comparison groups (851 up- and 672 down- regulated), 1993 DEGs in the 24 h groups (1119 up and 874 down) and 3074 DEGs in the 48 h groups (1862 up and 1212 down). In addition, the relationship between the DEGs of the three comparison groups was shown in a Venn diagram, and 149 genes were differentially expressed in all comparison groups (Figure 4C).

### 2.4. GO Function and KEGG Pathway Enrichment Analysis of DEGs

**Figure 5:**
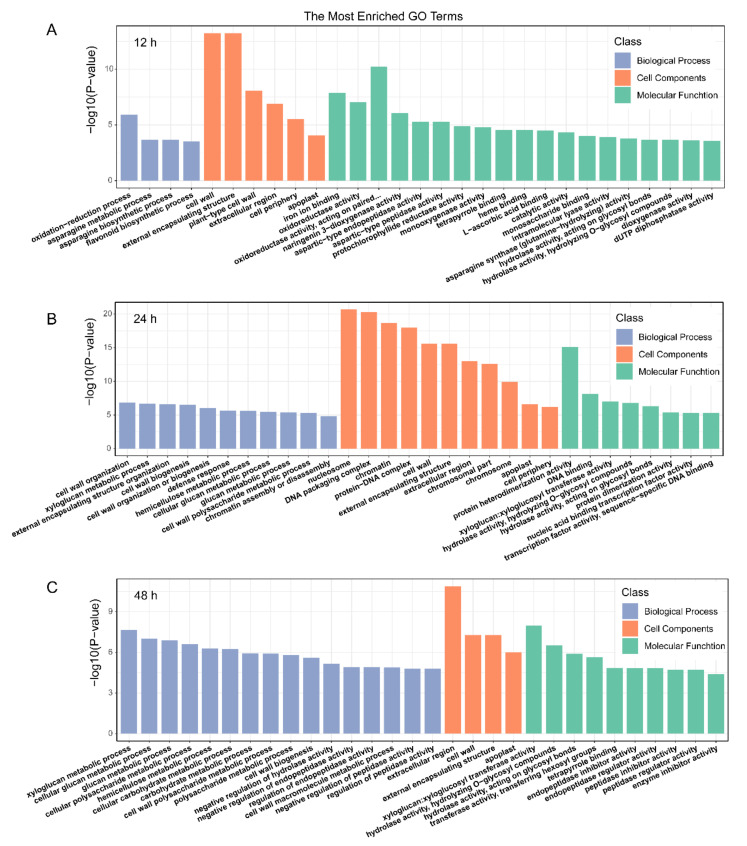
GO enrichment analysis of DEGs detected after treatment of leaves for 12 h (**A**), 24 h (**B**), and 48 h (**C**). The Top 30 GO terms with the most significant enrichment level are displayed in the order of *p*-value from small to large.

To identify the putative functions of the DEGs in three comparison groups, GO classification was performed. All the DEGs were divided into three categories, including biological process (BP), molecular function (MF) and cellular component (CC). In the GO annotation, 191, 268, and 236 terms were significantly enriched (*p* < 0.05) for the DEGs of 12 h, 24 h, and 48 h comparison groups, respectively. The most annotations of the level-two GO terms were “metabolic process”, “cellular process”, and “single-organism process” in the biological process, and cell”, “cell part”, “membrane” in the cellular component, and “binding”, and “catalytic activity” in molecular function. (Figure S1, Tables S3–S5). Furthermore, the most enriched GO terms are displayed in Figure 5. At 12 h, “oxidation-reduction process”, “asparagine metabolic process”, and “asparagine biosynthetic process” in BP, respectively, and “cell wall”, “external encapsulating structure”, and “plant-type cell wall” in CC, respectively, and “iron ion binding”, “oxidoreductase activity”, and “oxidoreductase activity” in MF, respectively, were the most enriched GO terms in their category. At 24 h, “cell wall organization”, “xyloglucan metabolic process”, and “external encapsulating structure organization” in BP, “nucleosome”, “DNA packaging complex”, and “chromatin” in CC, respectively, and “protein heterodimerization activity”, “DNA binding”, and “xyloglucan: xyloglucosyl transferase activity” in MF, respectively, were the most enriched GO terms in their category. At 48 h, “xyloglucan metabolic process”, “cellular glucan metabolic process”, and “glucan metabolic process” in BP, “extracellular region”, “cell wall”, and “external encapsulating structure” in CC, respectively, and “xyloglucan: xyloglucosyl transferase activity”, “hydrolase activity, hydrolyzing O-glycosyl compounds”, and “hydrolase activity, acting on glycosyl bonds” in MF, respectively, were the most enriched GO terms in their category.

**Figure 6:**
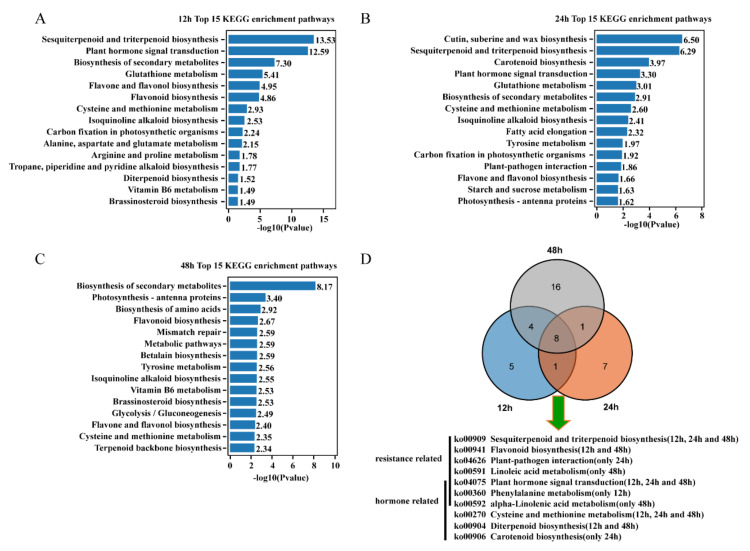
The KEGG enrichment pathway analysis of DEGs after treatment with 1′,4′-*trans*-diol-ABA. Top 15 significant pathways at 12 h (**A**), 24 h (**B**), and 48 h (**C**), respectively. Data were visualized using a bar plot with the *p*-value level indicated by ‘−log_10_(*p*value)’. (**D**) Venn diagram showing the overlap of common and unique pathways present in the transcriptome at 12, 24, and 48 h. The resistance- and hormone-related KEGG pathways for three time points is shown.

As the result of KEGG pathway enrichment analysis (Figure 6A–C, Tables S6–S8), The DEGs of 12 h group were significantly enriched (*p* < 0.05) 18 pathways, including sesquiterpenoid and triterpenoid biosynthesis, plant hormone signal transduction and the biosynthesis of secondary metabolites among others. In addition, 17 KEGG pathways were identified as significantly enriched in the 24 h group, including the biosynthesis of cutin, suberine and wax, sesquiterpenoid and triterpenoid, and carotenoid. Finally, the biosynthesis of secondary metabolites, photosynthesis-antenna proteins, and isoquinoline alkaloid biosynthesis were the most significant pathways of the total 29 significantly enriched pathways in the 48 h group. Of these enriched pathways, it was discovered that some hormone- and resistance-related pathways were enriched. In addition, the results showed that the plant hormone signal transduction, sesquiterpenoid and triterpenoid biosynthesis, and isoquinoline alkaloid biosynthesis pathways were enriched at all comparison groups (Figure 6D).

### 2.5. Transcription Factor Expression Analysis

**Figure 7:**
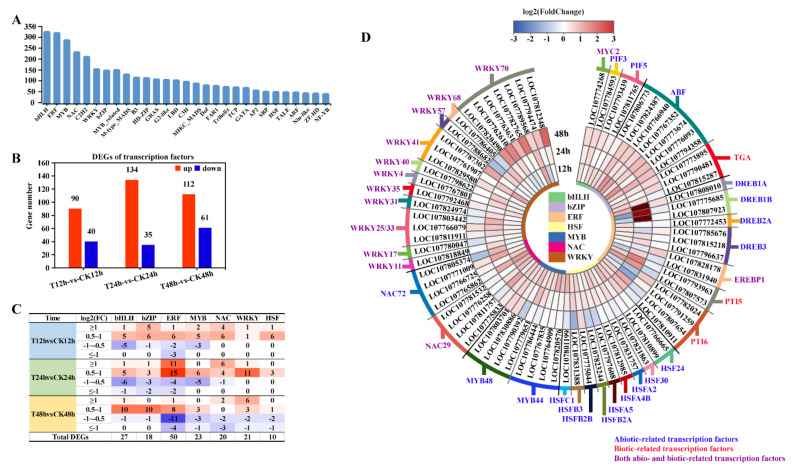
The expression of transcription factors that responded to 1′,4′-*trans*-diol-ABA treatment at different time points. (**A**)The TF families detected in tobacco; (**B**) The number of up- or down-regulated transcription factors. The statistical standard of DEGs was the FDR < 0.05, log_2_ (Fold Change) > 0.5 was up and log_2_ (Fold Change) < −0.5 was down. (**C**) The number of major TF family members classified according to the level of fold change; Red colors represent up-regulated, blue colors represent down-regulated; The darker the color, the more the number; (**D**) Heat-map differential expression of the TFs in the major families involved in the regulation of resistance to biotic or abiotic stresses. The inner circle colors represent the different families, and the outer circle colors represent specific transcripts. The color of gene name shows the different labeling about biotic and abiotic stress related TFs.

All the transcripts assembled in this study were compared with PlantTFDB database (Plant Transcription Factor Database) to predict the transcription factors (TFs). A total of 3626 TFs were annotated that belonged to 60 TF families (Table S9). The bHLH, ERF, MYB, NAC, and C2H2 families were the most abundant (Figure 7A). Expression profiling showed that there were 130 TFs (90 up-regulated and 40 down-regulated) at 12 h, 169 (134 up-regulated and 35 down-regulated) at 24 h and 173 (112 up-regulated and 61 down-regulated) at 48 h that were differentially expressed (Figure 7B).

The differential expression of key TF families closely related to plant disease resistance and stress adaption at different time points were analyzed, including the bHLH, bZIP, ERF, MYB, NAC, WRKY and HSF families. The results showed that more TF DEGs were up-regulated at 12 h and 24 h, whereas at 48 h, the number of up-regulated DEGs was approximately equal to that of the down-regulated DEGs (Figure 7C). The levels of expression of the TF DEGs involved in stress responses, which were primarily up-regulated, are shown in Figure 7D. In particular, the bHLH and bZIP family genes were generally up-regulated at three time points, particularly the ABFs and TGAs. In total, seven transcripts of ABF were detected in this study and all of them were up-regulated. In addition, three separate differential transcripts of TGA were up-regulated at two time points. Alternatively, the up-regulation of the DEGs including the ERF, HSF, MYB, NAC families primarily occurred at 12 h and 24 h. Three genes in the DREB subfamily of ERF were up-regulated more than 32-fold at 24 h (log_2_FC > 5). The patterns of expressions of the WRKY family members were varied, and most were up-regulated at 24 h. However, the expression of WRKY70 was generally up-regulated at 48 h, and this family contained the highest number of DEGs.

### 2.6. Analysis of Gene Expression in the ABA Signaling Pathway

**Figure 8:**
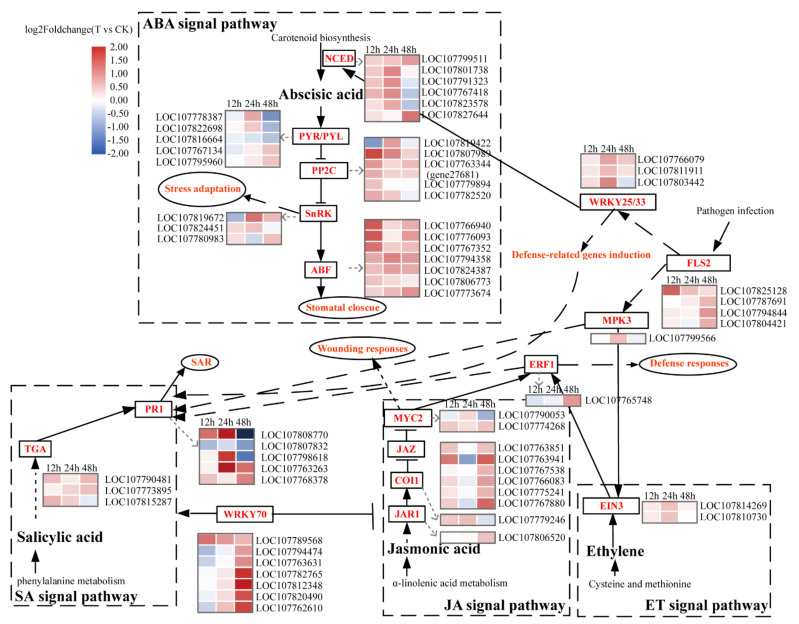
Different expression of key genes related to the ABA, SA, JA and ET signaling pathways. The heatmap data were log_2_ fold changes of DEGs that were significantly differentially expressed at least at one time point.

The KEGG enrichment results showed that the pathway related to hormones were enrichment at all 3 time points. Most of the hormone-related pathway involving DEGs were annotated in the ABA signaling pathway, including 21 non-redundant DEGs. The response of ABA-receptor PYR/PYL to 1′,4′-*trans*-diol-ABA appeared relatively later (without DEGs at 12 h). A total of five DEGs of all the 29 PYR/PYL genes were expressed separately at 24 h and 48 h, and six up-regulated DEGs were detected that belonged to the negative regulator PP2C. A total of three DEGs of SnRK2 were up-regulated. In addition, six 9-*cis*-epoxycaroterenoid dioxygenase (NCED) genes were primarily up-regulated. These results suggest that 1′,4′-*trans*-diol-ABA can induce the expression of genes involved in the ABA signaling pathway and also increase the expression of genes that regulate endogenous ABA synthesis (Table 1, Figure 8).

### 2.7. Other Hormone Signaling Pathway and Tolerance-Related Genes Expression Analysis

Further analyses of the expression of genes involved in salicylic acid (SA), jasmonic acid (JA) and ethylene (ET)pathways were performed (Table 1, Figure 8). A total of nine DEGs were identified from the SA pathway. In addition to the TGAs, four PR1-encoding DEGs were up-regulated at three time points but three were down-regulated at 48 h.

In the JA and ET signaling pathways, some regulatory genes presented differential patterns of expression after treatment with 1′,4′-*trans*-diol-ABA. Two EIN3 transcripts were up-regulated at 24 h in the ET pathway. The JAZ genes play key roles in the JA pathway, and two and six JAZ transcripts were found to be increased at 12 h and 48 h, respectively.

1′,4′-*trans*-diol-ABA also influenced the expressions of genes related enzymes in the reactive oxygen species (ROS) scavenging system to varying degrees. In particular, the genes encoding glutathione-S-transferases (GST) and glutaredoxin (GLR) were most remarkably induced. Most of 25 GST DEGs at 12 and 24 h were up-regulated. In addition, nine DEGs of GLRs were up-regulated at 48 h. In addition, 1′,4′-*trans*-diol-ABA had an effect on the expression of the genes that encoded catalase (CAT), peroxidase (POD), and superoxide dismutase (SOD) (Table 1).

Furthermore, some other genes related to stress tolerance were detected, including eight phenylalanine ammonia lyase (PAL) transcripts, 13 polyphenol oxidase (PPO) transcripts, 34 glutathione (GLU) transcripts and 34 chitinase transcripts. The influence on PALs was primarily inhibitory. The levels of expression of DEGs for PPOs were primarily up-regulated at 48 h. Two differentially expressed GLU transcripts were up-regulated at 48 h. In addition, six differently responsive transcripts of chitinase were primarily up-regulated, and four differentially expressed transcripts of leucine-rich repeat receptor kinase FLS2s were all up-regulated (Table 1).

### 2.8. Validation of RNA-Seq Data by a qRT-PCR Analysis

**Figure 9:**
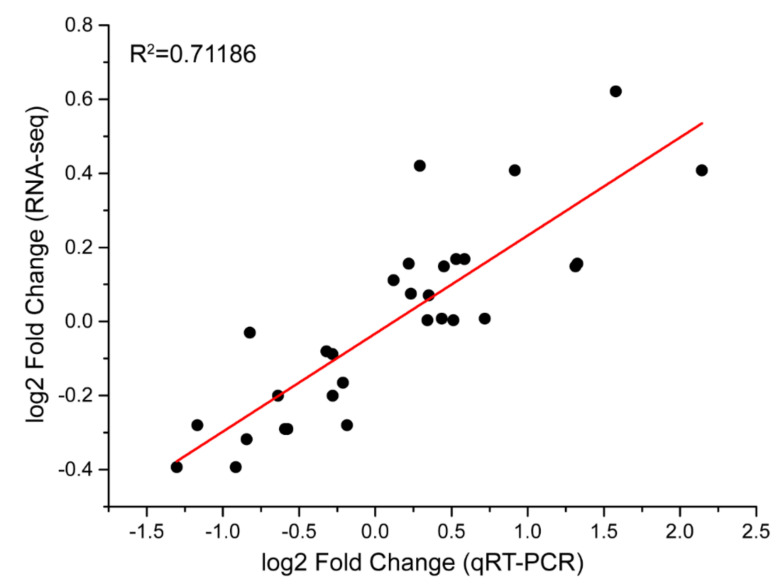
Correlation analysis of the selected genes based on qRT-PCR and RNA-seq data; Pearson’s correlation coefficient (R) was 0.84959 (*p* < 0.05), R^2^ = 0.71186.

A total of 10 genes were selected for a quantitative real-time reverse transcriptase-PCR (qRT-PCR) analysis to validate the quality of RNA-Seq data. As expected, most of these genes that were detected had similar tendencies for expression and were largely consistent with R^2^ = 0.71 (Pearson’s correlation coefficient) (Figure 9). The strong correlation between the RNA-Seq and qRT-PCR data indicates the reliability of the transcriptomic profiling data.

## 3. Discussion

Extensive studies have revealed that exogenous application of ABA induces the tolerance of a plant to a variety of abiotic stresses, such as drought, heat, cold, and high salinity [26,27,28], and some biotic stresses such as pathogens, and insects [29,30,31]. Many ABA analogues have been actively studied owing to their beneficial effects on plants [32,33]. Although there is no evidence to indicate that 1′,4′-*trans*-diol-ABA can be biosynthesized in plants, it has been confirmed as a precursor of ABA synthesis in microorganisms [13]. Its chemical structure is very similar to ABA, with the exception of the difference in chemical bonds at one site. However, the difficulty in obtaining pure samples significantly limit its research into stress tolerance or other activities as an analogue of ABA and related applications. Fortunately, a mutant strain of *B. cinerea* that can produce 1′,4′-*trans*-diol-ABA was isolated from fermentation broth in our laboratory, and a pure compound of more than 98% purity was obtained by purification methods developed in our laboratory. The acquisition of pure compound will provide a vital basis for the further study of this compound in anti-stress activities and efforts to elucidate its mechanism.

The phenotypic observations indicated that 2 mg/L 1′,4′-*trans*-diol-ABA induced stomata closure and enhanced the tolerance of tobacco to drought. Stomata have an important function in the response of plants to various abiotic stresses [34]. Under drought, the timely closure of stomata can effectively reduce water losses and prevent plants from being damaged owing to a lack of water [35]. The regulation of stomatal opening and closing is primarily mediated by endogenous ABA, as well as induction by a variety of exogenous ABA and stresses that occur as a result of adversity [36]. Similarly, 1′,4′-*trans*-diol-ABA triggered the closure of stomata to lessen the loss of water. Furthermore, we discuss the function of 1′,4′-*trans*-diol-ABA at the transcriptional level.

### 3.1. 1′,4′-trans-Diol-ABA Induced a Cascade Reaction of Gene Expression in Tobacco Leaves

Many molecular and physiological processes are reconfigured when plants are subjected to abiotic stress [37]. Numerous protective proteins and secondary metabolites are biosynthesized to help plants adapt to the environmental stresses by modulating a complex gene regulation network [38,39]. In this study, 1′,4′-*trans*-diol-ABA can change the expression of tobacco genes within a certain period of time, primarily by induction. After 1′,4′-*trans*-diol-ABA treatment, the DEGs with different functions varied in their response times, and the main enrichment pathways changed from hormonal signal transduction to the biosynthesis of small molecule and metabolites. These findings indicated that the expression of tobacco genes undergoes a cascade reaction [40].

### 3.2. 1′,4′-trans-Diol-ABA Induced the Expression of Transcription Factors Related to Stress Tolerance

The TFs interact with cis-elements in the promoter regions of several responsive genes and thus control the expression of many downstream genes. This enables them to trigger cascade reactions of many physiological processes and control biochemical reactions in plant cells [41]. Thus, a slight alteration in the transcript abundance of TFs can result in a dramatic change in downstream gene expression and physiological activities. Although a number of gene expression differences were less than 2-fold, as shown in the Figure 5C, they still cannot be ignored. Numerous members of multiple TF families are involved in the regulation of plant adverse tolerances in different forms (Figure 7D). In the NAC family, NAC29 can confer salt and drought stresses tolerance to wheat through the enhancement of antioxidant system and NAC72 can also improve the tolerance to plant drought stress [42,43]. MYB44 of the MYB family is involved in the regulation of expression of key genes in the ABA signal transduction pathway that enables its participation in the enhancement of stress tolerance [44]. Alternatively, DREBs are recognized as the subfamily involved in the regulation of various stresses, such as drought, high salt and low temperature, and this regulation from DERB1 and DREB2 has been reported as being independent of ABA signaling transduction [45]. Although the above-mentioned TFs participate in gene regulation by various ways during the process of plant stress responses, they were all induced by 1′,4′-*trans*-diol-ABA (Figure 7D). In addition, 1′,4′-*trans*-diol-ABA also improved the expression of many members of WRKY family, which is well-known for its regulation of not only abiotic stress tolerance but also pathogen resistance. WRKY70 is considered to regulate the SA and JA signaling pathways [46], and to enhance the tolerance of osmotic stress by regulating the ABA pathway to control the opening and closure of stomata [47]. WRKY25/33 has dual roles. They receive the biotic stress signal transmitted by the N gene to positively regulate the expression of the PR1 genes [48], and they positively regulate the expression of NCED, which is a key rate-limiting enzyme gene for ABA synthesis, and promotes the accumulation of ABA and enhances abiotic stress tolerance [49]. In our data, NCED genes were also detected that were induced by 1′,4′-*trans*-diol-ABA. This indicates that this ABA analogue may affect the homeostasis of ABA in plants and promote the synthesis of ABA to enhance plant stress tolerance. These findings suggests that the 1′,4′-*trans*-diol-ABA-mediated induction of TFs related to tolerance to biotic and abiotic stress would improve stress tolerance.

### 3.3. 1′,4′-trans-Diol-ABA Induced the ABA Signaling Involved Gene Expressions

The ABA signaling pathway is activated and regulated by the phytohormone ABA, and plays a key role in the regulation of plant stress tolerance [50]. A total of four key genes, the ABA receptor PYR/PYL, negative control factor PP2C, kinase SnRK2 and downstream TF ABF, participate in the regulation of ABA signaling. They are induced by ABA and stresses to transmit ABA signals that induce various reactions, such as an increase in ROS and the closure of stomata [19]. In this study, 1′,4′-*trans*-diol-ABA caused the up-regulation of many genes involved in the ABA signaling pathway, particularly the ABFs (Table 1, Figure 8). ABFs are inducible by abiotic stress, and there is both redundancy and specificity in their functions under various stress conditions. ABFs can interact with the coupling elements G-ABRE and CE3 to regulate the transcription of stress-responsive genes further promote stomatal closure and elevate survival rates under water-deficit conditions [51]. We found that all seven detected transcripts of ABF were induced, which provide additional powerful evidence at the transcription level that 1′,4′-*trans*-diol-ABA can induce tolerance.

### 3.4. Other Hormone-Related Genes Respond to 1′,4′-trans-Diol-ABA

Phytohormones regulate the normal growth and development of plants and respond to biotic and abiotic stresses [52].

In addition to the ABA signaling pathway that responded extensively to 1′,4′*trans*-diol-ABA, we also focused on the SA, JA, and ET signaling pathways (Table 1, Figure 8). The SA pathway plays an important role in plant defense, particularly in systemic acquired resistance (SAR). TGA and PR1 are the vital genes in SA pathway [53], which are primarily up-regulated. This finding suggested that 1′,4′-*trans*-diol-ABA may have a positive effect on SAR in the plant [53]. In addition, 1′,4′-*trans*-diol-ABA enhanced the levels of expression of multiple genes in the JA and ET signaling pathways. These results were consistent with the fact that exogenous ABA could induce the expression of SA, JA and ET pathway related genes [54]. Presumably, 1′,4′-*trans*-diol-ABA indirectly affected the hormone signals through its activation of the ABA signaling pathway, which may be related to the synergistic or antagonistic effects of various hormones in plant defense responses [55].

### 3.5. The Response of ROS Scavenging System and Other Defense-Related Genes to 1′,4′-trans-Diol-ABA

ROS play an important role in response of plants to stress and increases under various stresses. ROS have a dual function in plants, and their role transition depends on its concentration. ROS, as important signaling molecules in plants at low concentrations, participate in the regulation of plant growth and development and corresponding stress. Nevertheless, when their concentration becomes higher, ROS will affect the functions of protein, lipids and even nucleic acids, resulting in cell damage and even death [56]. Thus, there are a series of regulatory mechanisms to maintain the dynamic balance of ROS in plants. Our results showed that 1′,4′-*trans*-diol-ABA promotes the levels of expression of some of the genes in glutathione pathway that are involved the ROS scavenging system. Glutathione can effectively remove oxygen radicals in plants and maintain the dynamic balance of ROS in plant tissue [57]. Treatment with 1′,4′-*trans*-diol-ABA improves the expression of related genes, helps increase the activity of glutathione and can effectively prevent or alleviate the damage of ROS to plants caused by adversity.

Other genes related to both biotic and abiotic stress responses were affected by 1′,4′-*trans*-diol-ABA, particularly PAL, PPO and GLU among others. Alternatively, FLS2 and chitinase play important roles in identifying and defending against pathogens in plant immunity [58]. These up-regulated genes could promote defense against pathogens. In addition, stomatal closure has a positive function in innate immunity against bacterial invasion [59]. Thus, these results suggest that 1′,4′-*trans*-diol-ABA has positive effects on plant biotic defense.

**Figure 10:**
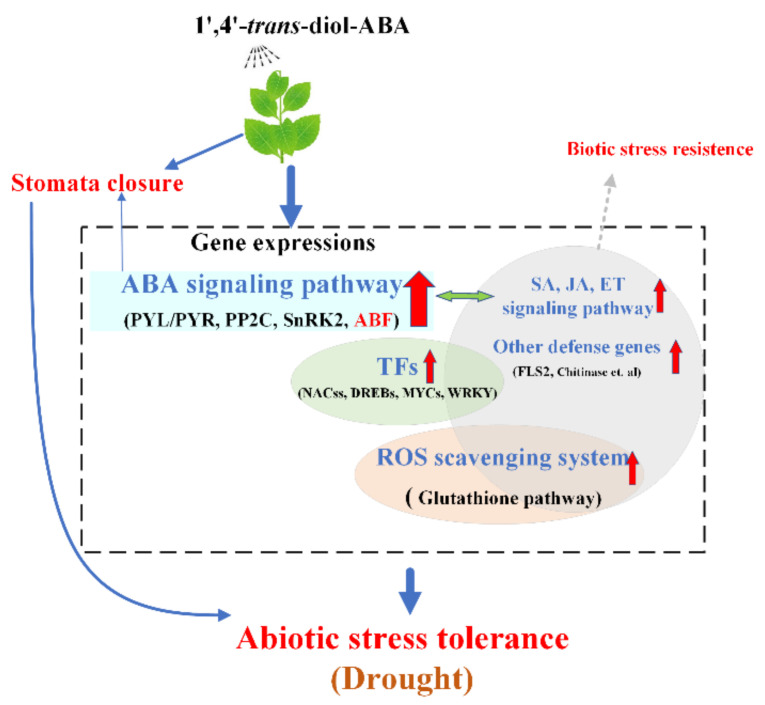
The summary about 1′,4′-*trans*-diol-ABA inducing stress tolerance by affecting the level of gene expressions in tobacco. The red upward arrows indicate that the genes are up-regulated expressing.

Consequently, 1′,4′-*trans*-diol-ABA can regulate gene expressions to cope with drought and other stresses in tobacco (Figure 10).

## 4. Conclusions

This study revealed that 1′,4′-*trans*-diol-ABA can improve drought stress tolerance and close leaf stomata, as well as having a profound influence on the global transcription level of plant genes. A large number of genes related to abiotic and biotic tolerance were identified as being induced by this compound, particularly the ABA signaling pathway, the transcription factors of NAC, DREB, MYB, WRKY, and ROS scavenging system and some other defense genes. All these findings indicate 1′,4′-*trans*-diol-ABA has the potential to enhance plant tolerance to abiotic stresses ad resistance to disease. This research provides a foundation for the further exploration of potential functions and the application in agriculture.

## 5. Materials and Methods

### 5.1. Plant Materials and Treatment

1′,4′-*trans*-diol-ABA was extracted using our reported method [17]: The mutant strain of *B. cinerea* was grown in the self-made medium for fifteen days; The compound in fermentation broth was obtained by macroreticular resin adsorption, desorption and crystallization; then, the needle crystal was obtained by MeOH-recrystallization (purity ≥ 98%) (Figure S2). This compound has a molecular formula of C_15_H_22_O_4_ based on its HR-ESI-MS at *m/z* 265.1446 [M-H]^−^, the detailed information about the HR-ESI-MS and nuclear magnetic resonance (NMR) results of 1′,4′-trans-diol-ABA were shown by Supplementary File S1. All these results are consistent with our previous report [16]. The crystal structure of 1′,4′-*trans*-diol-ABA was determined through X-ray diffraction analysis by ENRAF NONIUS CAD4 diffractometer [17]. The compound was dissolved in 95% ethanol to prepare a stock solution of 4000 mg/L. The working concentration was diluted with water. The same proportion of ethanol was added to the water used for the control.

Tobacco (*Nicotiana tabacum* L.) Seeds of cv. Hong Hua Da Jin Yuan were planted and grown in plastic pots with organic loam in March, 2019, and grown in a greenhouse in Shifang, Sichuan Province, China (104° E, 31° N). The sunshine is approximately 13 h per day. The temperature was controlled at 26 and 20 °C at day and night, respectively.

In a preliminary experiment, the concentration of 1′,4′-*trans*-diol-ABA to apply was optimized, and the results showed that a more effective anti-stress effect could be achieved when the concentration reached 2 mg/L. Therefore, this concentration was used for these studies.

When the tobacco seedlings grown to 2 months old, they were treated. For the drought tolerance treatments, after three days without watering, leaf surfaces of treatment seedlings (T) and drought control (D) were sprayed with 2 mg/L (7.52 μmol/L) 1′,4′-*trans*-diol-ABA and an equal amount of water, respectively. Then water supply was periodically stopped after treatment. The changes in tobacco seedlings under drought stress were observed, and the blank control (CK) was watered every two days. After an observation period, the aboveground parts of the plant were dried to measure dry matter quality and the number of leaves were counted per experiment. Comparisons between different treated groups were assessed using two-way ANOVA analysis with SPSS 20 software (IBM, USA).

To prepare the samples for RNA-Seq, the whole plant was sprayed with 2 mg/L (7.52 μmol/L) 1′,4′-*trans*-diol-ABA, and the control was sprayed with the same amount of pure water. The 3rd to 5th euphylla samples of the control (CK) and treatment groups (T) were collected at 12 h, 24 h, and 48 h after treatment. Each three plants were mixed with three replicates for each sample, which were stored in liquid nitrogen.

For the blade stomatal movement trial, the sterilized seeds were spotted on 1/2 MS solid media. After 24 h of dark culture, the seeds were transferred to a light incubator with 16 h light for 12–14 days at a relative humidity of 60%, temperature of 25 ± 1 °C, and a light intensity of 3000 Lx. The leaves were torn off at the epidermis, placed in sterilized water, treated with 2 mg/L 1′,4′-*trans*-diol-ABA, and 2 mg/L ABA, respectively, and incubated in light for 0.5 h and 1 h. The stomata were observed and recorded under optical microscopy (Nikon, Eclipse E200, Tokyo, Japan). The experiment was repeated three times. ImageJ software (National Institutes of Health, Bethesda, MD, USA) was used to determine the aperture of 40 stomata per experiment. Comparisons statistical analysis was same as above.

### 5.2. RNA Isolation and Transcriptome Sequencing

The Total RNA of 18 leaf samples was isolated using an RNAiso Plus Reagent (TaKaRa, Beijing, China) according to the manufacturer’s instructions. The concentration and quality of the RNA samples were determined using a NanoDrop spectrophotometer (Thermo Scientific, Waltham, MA, USA). The RNA Integrity was determined by 1% agarose gel electrophoresis and an Agilent 2100 Bioanalyzer (Agilent Technologies, Santa Clara, CA, USA). Sequencing libraries were generated using a TruSeq RNA Sample Preparation Kit (Illumina, San Diego, CA, USA).

In this study, oligo (dT) magnetic beads were used to enrich mRNA with a polyA structure in the total RNA, and the RNA was disrupted to 200–300 bp fragments using ion disruption. The first strand of cDNA was synthesized using a 6-base random primer and reverse transcriptase, and the second strand cDNA was synthesized using the first strand cDNA as a template. After the library construction was complete, the library fragments were enriched by PCR amplification, and library selection was performed according to fragment size. The size of library was 300–400 bp. Next, the library was quality tested using an Agilent 2100 Bioanalyzer and the total and effective concentrations of the library were examined. Samples were subjected to RNA extraction and purification for library construction, and these libraries were paired-end (PE) sequenced based on an Illumina HiSeq sequencing platform. The RNA library construction was conducted by Nanjing Personal Bioinformatics Co., Ltd. (Nanjing, China). The RNA-Seq data were submitted to the NCBI database with the SRA accession number PRJNA684346.

### 5.3. Raw Data Sorting, Filtering, and Quality Evaluation

After sequencing, FASTQ raw data is generated, and the data is statistically analyzed such as Q20 (%) and Q30 (%). The reads with adapters and the low-quality reads were filtered in the sequencing data: Cutadapt was used to remove the 3′-end adapter sequences; Reads with an average mass fraction lower than Q20 were removed.

### 5.4. Reference Genome Comparison and Quantification of Expression Level

The HISAT2 version 2.1.0 (https://daehwankimlab.github.io/hisat2/, accessed on 4 March 2021) software [60], which the upgraded version of TopHat2 [61], was used to compare the filtered Reads to the reference genome (tobacco TN90, GCF_000715135.1, NCBI) [62]. The distribution of Reads compared to the genome was counted, and the mapping regions were divided into CDS (coding region), Intron (intron), Intergenic (intergenic region), and UTR (5′ and 3′ untranslated regions). The number of reads that mapped to each gene were obtained using HTSeq-count software [63]. The reads count is positively related to the true expression level of the gene, as well as the length of the gene and the sequencing depth. The Fragments Per Kilobase per Million (FPKM) [64] were then used to determine the relative level of expression of each gene.

### 5.5. Differentially Expressed Gene Analysis and Functional Enrichment

In order to eliminate the influence of rhythm and growth as far as possible, the comparison between the treatment and the control groups was carried out at the same time point. Settings of differentially expressed gene analysis groups were compared and divided into groups T12 h vs CK12 h, T24 h vs CK24 h, T48 h vs CK48 h. DEGs were determined based on the adjusted read counts of each transcript in a pair of libraries using the edgeR (Empirical Analysis of Digital Gene Expression Data in R) package [65]. The edgeR is a widely recognized and used Bioconductor software package for examining differential expression of replicated count data [66,67]. It is used for differential expression analysis of RNA-seq expression profiles with biological replication. It implements a range of statistical methodology based on the negative binomial distributions, including empirical Bayes estimation, exact tests, generalized linear models and quasi-likelihood tests [65,68]. Here, the expression level of a DEG was declared to be significant if the FDR was < 0.05 and a |log_2_ (Fold Change)| > 0.5 was observed.

After data correction using the R package, TBtools software [69] was used to determine the statistical enrichment of DEGs in Gene Ontology (GO, http://geneontology.org/, accessed on 4 March 2021) and Kyoto Encyclopedia of Gene and Genome (KEGG, http://www.genome.jp/kegg/, accessed on 4 March 2021) using an adjusted *p*-value ≤ 0.05 to determine a significantly enriched pathway.

### 5.6. qRT-PCR Verification

A total of ten genes were randomly selected for validation by qPCR. RNA was extracted from the leaves of independent biological replicates for each of CK12 h, CK24 h, CK48 h, T12 h, T24 h and T48 h were employed for qPCR validation. The PrimeScript^TM^ RT reagent Kit with gDNA Eraser (Perfect Real Time) (TaKaRa) was used to remove the gDNA and synthesize cDNA as the template for qPCR. Gene copy specific primers for qPCR were designed based on the corresponding sequence on Primer Premier 5 software (Table S9) and synthesized by TSINGKE (Chengdu, China). The reaction was performed on CFX96 Touch Real-Time PCR Detection System (Bio-Rad, Hercules, CA, USA). The program began at 95 °C for 5 min, followed by 40 cycles of 95 °C for 10 s and 55 °C for 30 s. The reference gene EF-1α (LOC107826390) was used as an internal control [70]. The 2^−ΔΔ*C*t^ method was used to evaluate the relative levels of gene expression [71]. The relative gene expression data of qPCR and the differential expression data of RNA-Seq were combined for regression analysis by Origin software (OriginLab, Northampton, MA, USA) to verify the results of gene expression of RNA-Seq.

## Figures and Tables

**Table 1:** Differential expression of the genes related to ABA, jasmonic acid, salicylic acid, and ethylene signaling pathways, reactive oxygen species scavenging, and other stress tolerance related genes in the tobacco transcriptome.

Trait	Description	12 h	24 h	48 h	Total DEGs	Sum
		up(down) *	up(down)	up(down)		
**Abscisic Acid**	NCED	2(0)	5(0)	2(2)	6	6
	PYR/PYL	0(0)	1(0)	2(3)	5	21
	PP2C	5(0)	2(0)	1(0)	6	13
	SnRK2	0(1)	2(0)	2(0)	3	19
	ABF	6(0)	3(0)	6(0)	7	7
**Salicylic Acid**	TGA	2(0)	0(0)	2(0)	3	9
	PR1	1(0)	3(0)	2(3)	5	5
**Jasmonic Acid**	JAR1	0(0)	0(0)	1(0)	1	7
	COI1	0(0)	1(0)	0(0)	1	4
	JAZ	2(0)	0(1)	6(0)	6	9
	MYC2	0(0)	0(0)	1(1)	4	9
**Ethylene**	EIN3	0(0)	2(0)	0(0)	2	11
	ERF1	0(0)	0(0)	0(1)	1	2
**ROS Scavenging System**	CAT	0(0)	1(1)	0(1)	2	6
	POD	2(2)	4(2)	5(5)	14	34
	SOD	1(0)	2(0)	0(0)	3	16
	GST	14(2)	9(3)	3(9)	25	54
	GLR	1(1)	1(1)	9(0)	12	40
	APX	0(1)	0(0)	1(1)	2	15
	Trx	0(0)	2(4)	1(1)	6	30
	PrxR	0(1)	0(0)	0(0)	1	9
**Other Stress Tolerance Related Genes**	PAL	0(1)	0(1)	0(2)	3	8
	PPO	1(1)	1(3)	5(1)	6	6
	GLU	0(0)	2(0)	0(0)	2	2
	FLS2	1(0)	1(0)	3(0)	4	7
	chitinase	3(0)	1(0)	3(1)	6	15

ABF, ABA-responsive element binding factor; APX ascorbate peroxidase; CAT, catalase; COI1, coronatine-insensitive protein 1; EIN3, ethylene-insensitive protein 3; ERF1, ethylene-responsive element-binding factor 1; FLS2, flagellin-sensing 2; GLR, glutaredoxin; GLU, beta-1,3-glucanase; GST, glutathione-S-transferase; JAR1, jasmonate resistant 1; JAZ, jasmonate ZIM-domain protein; MYC2, Myelocytomatosis proteins 2; NCED, 9-*cis*-epoxycaroterenoid dioxygenase; PAL, phenylalanine ammonia-lyase; POD, peroxidase; PP2C, phosphatase 2C; PPO, polyphenol oxidase; PR1, pathogenesis-related protein 1; PrxR, peroxiredoxin; PYR/PYL, pyrabactin resistance1/PYR1-like; SnRK2, sucrose non-fermenting 1-related protein kinase 2; SOD, superoxide dismutase; TGA, TGAGG motif-binding factor; Trx, thioredoxin. * The statistical standard of DEGs was the FDR < 0.05, log_2_ (fold change) > 0.5 was up and log_2_ (fold change) < −0.5 was down.

## Data Availability

The data used in this study can be obtained from the corresponding author on reasonable request.

## Supplementary Materials

The following are available online at https://www.mdpi.com/1422-0067/22/5/2555/s1, Figure S1: Level 2 GO term categorization of DEGs at 12 h (A), 24 h (B), and 48 h (C). The red bar is up-regulated number of DEGs, and the blue is down-regulated. Figure S2: The crystal of 1′,4′-*trans*-diol-ABA. Table S1: Summary of RNA sequencing statistics from tobacco leaves of 1′,4′-*trans*-diol-ABA and control sampled at 12 h, 24 h and 48 h after treated; Table S2: Gene differential expression Analysis at 12 h, 24 h and 48 h between treatment group and control group; Table S3: Significant GO-term enrichment of 12 h DEGs; Table S4: Significant GO-term enrichment of 24 h DEGs; Table S5: Significant GO-term enrichment of 48 h DEGs; Table S6: Significant KEGG pathway analysis of 12 h DEGs; Table S7: Significant KEGG pathway analysis of 24 h DEGs; Table S8: Significant KEGG pathway analysis of 48 h DEGs; Table S9: Transcription factor family detail; Table S10: Primers for qPCR. Supplementary File S1: The results about HR-ESR-MS and NMR of 1′,4′-*trans*-diol-ABA.

## References

[1] Rivas-San Vicente M., Plasencia J. (2011). Salicylic acid beyond defence: Its role in plant growth and development. J. Exp. Bot..

[2] Raghavendra A.S., Gonugunta V.K., Christmann A., Grill E. (2010). ABA perception and signalling. Trends Plant Sci..

[3] Finkelstein R.R., Gampala S.S., Rock C.D. (2002). Abscisic acid signaling in seeds and seedlings. Plant Cell.

[4] Fujita Y., Fujita M., Shinozaki K., Yamaguchi-Shinozaki K. (2011). ABA-mediated transcriptional regulation in response to osmotic stress in plants. J. Plant Res..

[5] Chinnusamy V., Gong Z., Zhu J.K. (2008). Abscisic acid-mediated epigenetic processes in plant development and stress responses. J. Integr. Plant Biol..

[6] Sun Y., Pri-Tal O., Michaeli D., Mosquna A. (2020). Evolution of Abscisic Acid Signaling Module and Its Perception. Front Plant Sci..

[7] Walton D.C., Sondheimer E. (1972). Activity and metabolism of C-(+/−)-abscisic Acid derivatives. Plant Physiol..

[8] Siewers V., Kokkelink L., Smedsgaard J., Tudzynski P. (2006). Identification of an abscisic acid gene cluster in the grey mold Botrytis cinerea. Appl. Environ. Microbiol..

[9] Hirai N. (1986). The 1′,4′-trans-diol of abscisic acid, a possible precursor of abscisic acid in Botrytis cinerea. Phytochemistry.

[10] Neill S., Horgan R., Walton D., Mercer C. (1987). Biosynthesis of ABA in C-rosicola. 4. The Metabolism Of Alpha-Ionylidene Compounds By Cercospora-Rosicola. Phytochemistry.

[11] Okamoto M., Hirai N., Koshimizu K. (1988). Biosynthesis of abscisic acid in Cercospora pini-densiflorae. Phytochemistry.

[12] Okamoto M., Hirai N., Koshimizu K. (1988). Biosynthesis of abscisic acid from α-ionylideneethanol in Cercospora pini-densiflorae. Phytochemistry.

[13] Milborrow B.V., Lee H.S. (1998). Endogenous biosynthetic precursors of (+)-abscisic acid. VII. The 1′,4′-trans-diol is formed from ABA, it is not a precursor. Funct. Plant Biol..

[14] Rock C.D., Zeevaart J.A.D. (1990). Abscisic (ABA)-Aldehyde Is a Precursor to, and 1′,4′-trans-ABA-Diol a Catabolite of, ABA in Apple. Plant Physiol..

[15] Sindhu R.K., Walton D.C. (1988). Xanthoxin Metabolism in Cell-free Preparations from Wild Type and Wilty Mutants of Tomato. Plant Physiol..

[16] Wang T., Zhou J., Bai B., Yang J., Tan H. (2005). Stereochemistry And Biological Activity of 1′,4′-trans-diol of ABA. Chin. J. Appl. Environ. Biol..

[17] Wang T.S., Zhou J.Y., Tan H. (2006). Isolation and Crystal Structure of 1′,4′-Trans-diol of Abscisic Acid. Chin. J. Struc. Chem..

[18] Finkelstein R. (2013). Abscisic Acid synthesis and response. Arab. Book.

[19] Ali A., Pardo J.M., Yun D.J. (2020). Desensitization of ABA-Signaling: The Swing From Activation to Degradation. Front. Plant Sci..

[20] Cutler S.R., Rodriguez P.L., Finkelstein R.R., Abrams S.R. (2010). Abscisic Acid: Emergence of a Core Signaling Network. Annu. Rev. Plant Biol..

[21] Zhao Q., Hu R.S., Liu D., Liu X., Wang J., Xiang X.H., Li Y.Y. (2020). The AP2 transcription factor NtERF172 confers drought resistance by modifying NtCAT. Plant Biotechnol. J..

[22] Zhou S., Zheng W.J., Liu B.H., Zheng J.C., Dong F.S., Liu Z.F., Wen Z.Y., Yang F., Wang H.B., Xu Z.S. (2019). Characterizing the Role of TaWRKY13 in Salt Tolerance. Int. J. Mol. Sci..

[23] Hoang X.L.T., Nguyen N.C., Nguyen Y.H., Watanabe Y., Tran L.P., Thao N.P. (2019). The Soybean GmNAC019 Transcription Factor Mediates Drought Tolerance in Arabidopsis in an Abscisic Acid-Dependent Manner. Int. J. Mol. Sci..

[24] Zhang B., Su L., Hu B., Li L. (2018). Expression of AhDREB1, an AP2/ERF Transcription Factor Gene from Peanut, Is Affected by Histone Acetylation and Increases Abscisic Acid Sensitivity and Tolerance to Osmotic Stress in Arabidopsis. Int. J. Mol. Sci..

[25] Abe H., Urao T., Ito T., Seki M., Shinozaki K., Yamaguchi-Shinozaki K. (2003). Arabidopsis AtMYC2 (bHLH) and AtMYB2 (MYB) function as transcriptional activators in abscisic acid signaling. Plant Cell.

[26] Wang H., Blakeslee J.J., Jones M.L., Chapin L.J., Dami I.E. (2020). Exogenous abscisic acid enhances physiological, metabolic, and transcriptional cold acclimation responses in greenhouse-grown grapevines. Plant Sci..

[27] Wu X., Liang C.J. (2017). Enhancing tolerance of rice (Oryza sativa) to simulated acid rain by exogenous abscisic acid. Environ. Sci. Pollut. R..

[28] An Y., Zhou P., Liang J.F. (2014). Effects of exogenous application of abscisic acid on membrane stability, osmotic adjustment, photosynthesis and hormonal status of two lucerne (*Medicago sativa* L.) genotypes under high temperature stress and drought stress. Crop Pasture Sci..

[29] Atkinson N.J., Urwin P.E. (2012). The interaction of plant biotic and abiotic stresses: From genes to the field. J. Exp. Bot..

[30] Liu J.L., Du H.T., Ding X., Zhou Y.D., Xie P.F., Wu J.C. (2017). Mechanisms of callose deposition in rice regulated by exogenous abscisic acid and its involvement in rice resistance to Nilaparvata lugens Stal (Hemiptera: Delphacidae). Pest Manag. Sci..

[31] Song W., Ma X., Tan H., Zhou J. (2011). Abscisic acid enhances resistance to Alternaria solani in tomato seedlings. Plant Physiol. Biochem. Ppb.

[32] Vaidya A.S., Helander J.D.M., Peterson F.C., Elzinga D., Cutler S.R. (2019). Dynamic control of plant water use using designed ABA receptor agonists. Science.

[33] Cao M.J., Zhang Y.L., Liu X., Huang H., Zhou X.E., Wang W.L., Zeng A., Zhao C.Z., Si T., Du J. (2017). Combining chemical and genetic approaches to increase drought resistance in plants. Nat. Commun..

[34] Neill S., Barros R., Bright J., Desikan R., Hancock J., Harrison J., Morris P., Ribeiro D., Wilson I. (2008). Nitric oxide, stomatal closure, and abiotic stress. J. Exp. Bot.

[35] Hajihashemi S. (2019). Stomatal Regulation as a Drought-tolerance Mechanism.

[36] Wilkinson S., Davies W.J. (2002). ABA-based chemical signalling: The co-ordination of responses to stress in plants. Plant Cell Environ..

[37] Hirayama T., Shinozaki K. (2010). Research on plant abiotic stress responses in the post-genome era: Past, present and future. Plant J..

[38] Heidarvand L., Amiri R.M. (2010). What happens in plant molecular responses to cold stress?. Acta Physiol. Plant..

[39] Zhu J., Dong C.H., Zhu J.K. (2007). Interplay between cold-responsive gene regulation, metabolism and RNA processing during plant cold acclimation. Curr. Opin. Plant Biol..

[40] Cramer G.R., Urano K., Delrot S., Pezzotti M., Shinozaki K. (2011). Effects of abiotic stress on plants: A systems biology perspective. BMC Plant Biol..

[41] Ramirez S.R., Basu C. (2009). Comparative Analyses of Plant Transcription Factor Databases. Curr. Genom..

[42] Huang Q., Wang Y., Li B., Chang J., Chen M., Li K., Yang G., He G. (2015). TaNAC29, a NAC transcription factor from wheat, enhances salt and drought tolerance in transgenic Arabidopsis. BMC Plant Biol..

[43] Tran L.S.P., Nakashima K., Sakuma Y., Simpson S.D., Fujita Y., Maruyama K., Fujita M., Seki M., Shinozaki K., Yamaguchi-Shinozaki K. (2004). Isolation and functional analysis of Arabidopsis stress-inducible NAC transcription factors that bind to a drought-responsive cis-element in the early responsive to dehydration stress 1 promoter. Plant Cell.

[44] Jung C., Seo J.S., Han S.W., Koo Y.J., Kim C.H., Song S.I., Nahm B.H., Choi Y.D., Cheong J.J. (2008). Overexpression of AtMYB44 enhances stomatal closure to confer abiotic stress tolerance in transgenic Arabidopsis. Plant Physiol..

[45] Yoshida T., Mogami J., Yamaguchi-Shinozaki K. (2014). ABA-dependent and ABA-independent signaling in response to osmotic stress in plants. Curr. Opin. Plant Biol..

[46] Li J., Brader G., Kariola T., Palva E.T. (2006). WRKY70 modulates the selection of signaling pathways in plant defense. Plant J..

[47] Li J., Besseau S., Toronen P., Sipari N., Kollist H., Holm L., Palva E.T. (2013). Defense-related transcription factors WRKY70 and WRKY54 modulate osmotic stress tolerance by regulating stomatal aperture in Arabidopsis. New Phytol..

[48] Zheng Z., Qamar S.A., Chen Z., Mengiste T. (2006). Arabidopsis WRKY33 transcription factor is required for resistance to necrotrophic fungal pathogens. Plant J..

[49] Luo D.L., Ba L.J., Shan W., Kuang J.F., Lu W.J., Chen J.Y. (2017). Involvement of WRKY Transcription Factors in Abscisic-Acid-Induced Cold Tolerance of Banana Fruit. J. Agric. Food Chem..

[50] Nakashima K., Yamaguchi-Shinozaki K. (2013). ABA signaling in stress-response and seed development. Plant Cell Rep..

[51] Kim S.Y. (2010). The role of ABF family bZIP class transcription factors in stress response. Physiol. Plant..

[52] Robert-Seilaniantz A., Grant M., Jones J.D. (2011). Hormone crosstalk in plant disease and defense: More than just jasmonate-salicylate antagonism. Annu. Rev. Phytopathol..

[53] Gao Q.M., Zhu S., Kachroo P., Kachroo A. (2015). Signal regulators of systemic acquired resistance. Front. Plant Sci..

[54] Wang Y., Tao X., Tang X.M., Xiao L., Sun J.L., Yan X.F., Li D., Deng H.Y., Ma X.R. (2013). Comparative transcriptome analysis of tomato (Solanum lycopersicum) in response to exogenous abscisic acid. BMC Genom..

[55] Bari R., Jones J.D. (2009). Role of plant hormones in plant defence responses. Plant Mol. Biol..

[56] Mittler R., Vanderauwera S., Gollery M., Van Breusegem F. (2004). Reactive oxygen gene network of plants. Trends Plant Sci..

[57] Gill S.S., Tuteja N. (2010). Reactive oxygen species and antioxidant machinery in abiotic stress tolerance in crop plants. Plant Physiol. Biochem..

[58] Robatzek S., Wirthmueller L. (2013). Mapping FLS2 function to structure: LRRs, kinase and its working bits. Protoplasma.

[59] Melotto M., Underwood W., Koczan J., Nomura K., He S.Y. (2006). Plant stomata function in innate immunity against bacterial invasion. Cell.

[60] Kim D., Paggi J.M., Park C., Bennett C., Salzberg S.L. (2019). Graph-based genome alignment and genotyping with HISAT2 and HISAT-genotype. Nat. Biotechnol..

[61] Kim D., Pertea G., Trapnell C., Pimentel H., Kelley R., Salzberg S.L. (2013). TopHat2: Accurate alignment of transcriptomes in the presence of insertions, deletions and gene fusions. Genome Biol..

[62] Sierro N., Battey J.N., Ouadi S., Bakaher N., Bovet L., Willig A., Goepfert S., Peitsch M.C., Ivanov N.V. (2014). The tobacco genome sequence and its comparison with those of tomato and potato. Nat. Commun..

[63] Anders S., Pyl P.T., Huber W. (2015). HTSeq--a Python framework to work with high-throughput sequencing data. Bioinformatics.

[64] Trapnell C., Williams B.A., Pertea G., Mortazavi A., Kwan G., van Baren M.J., Salzberg S.L., Wold B.J., Pachter L. (2010). Transcript assembly and quantification by RNA-Seq reveals unannotated transcripts and isoform switching during cell differentiation. Nat. Biotechnol..

[65] Robinson M.D., McCarthy D.J., Smyth G.K. (2010). edgeR: A Bioconductor package for differential expression analysis of digital gene expression data. Bioinformatics.

[66] Pattison R.J., Csukasi F., Zheng Y., Fei Z., van der Knaap E., Catala C. (2015). Comprehensive Tissue-Specific Transcriptome Analysis Reveals Distinct Regulatory Programs during Early Tomato Fruit Development. Plant Physiol..

[67] Liu J., Shi M., Wang J., Zhang B., Li Y., Wang J., El-Sappah A.H., Liang Y. (2020). Comparative Transcriptomic Analysis of the Development of Sepal Morphology in Tomato (*Solanum Lycopersicum* L.). Int. J. Mol. Sci..

[68] McCarthy D.J., Chen Y., Smyth G.K. (2012). Differential expression analysis of multifactor RNA-Seq experiments with respect to biological variation. Nucleic Acids Res..

[69] Chen C., Chen H., Zhang Y., Thomas H.R., Frank M.H., He Y., Xia R. (2020). TBtools—An integrative toolkit developed for interactive analyses of big biological data. Mol. Plant.

[70] Schmidt G.W., Delaney S.K. (2010). Stable internal reference genes for normalization of real-time RT-PCR in tobacco (Nicotiana tabacum) during development and abiotic stress. Mol. Genet. Genom..

[71] Livak K.J., Schmittgen T.D. (2001). Analysis of relative gene expression data using real-time quantitative PCR and the 2(T)(-Delta Delta C) method. Methods.

