# Supplementary material for: Exogenous 1′,4′-trans-Diol-ABA Induces Stress Tolerance by Affecting the Level of Gene Expression in Tobacco (Nicotiana tabacum L.)
Source: Int J Mol Sci. 2021 Mar 4;22(5):2555. doi: 10.3390/ijms22052555 (PMC7961390; doi:10.3390/ijms22052555)
Supplement: Supplementary file 1 [file ijms-22-02555-s001.zip › ijms-1085705-supplementary/Figure S1.pdf]

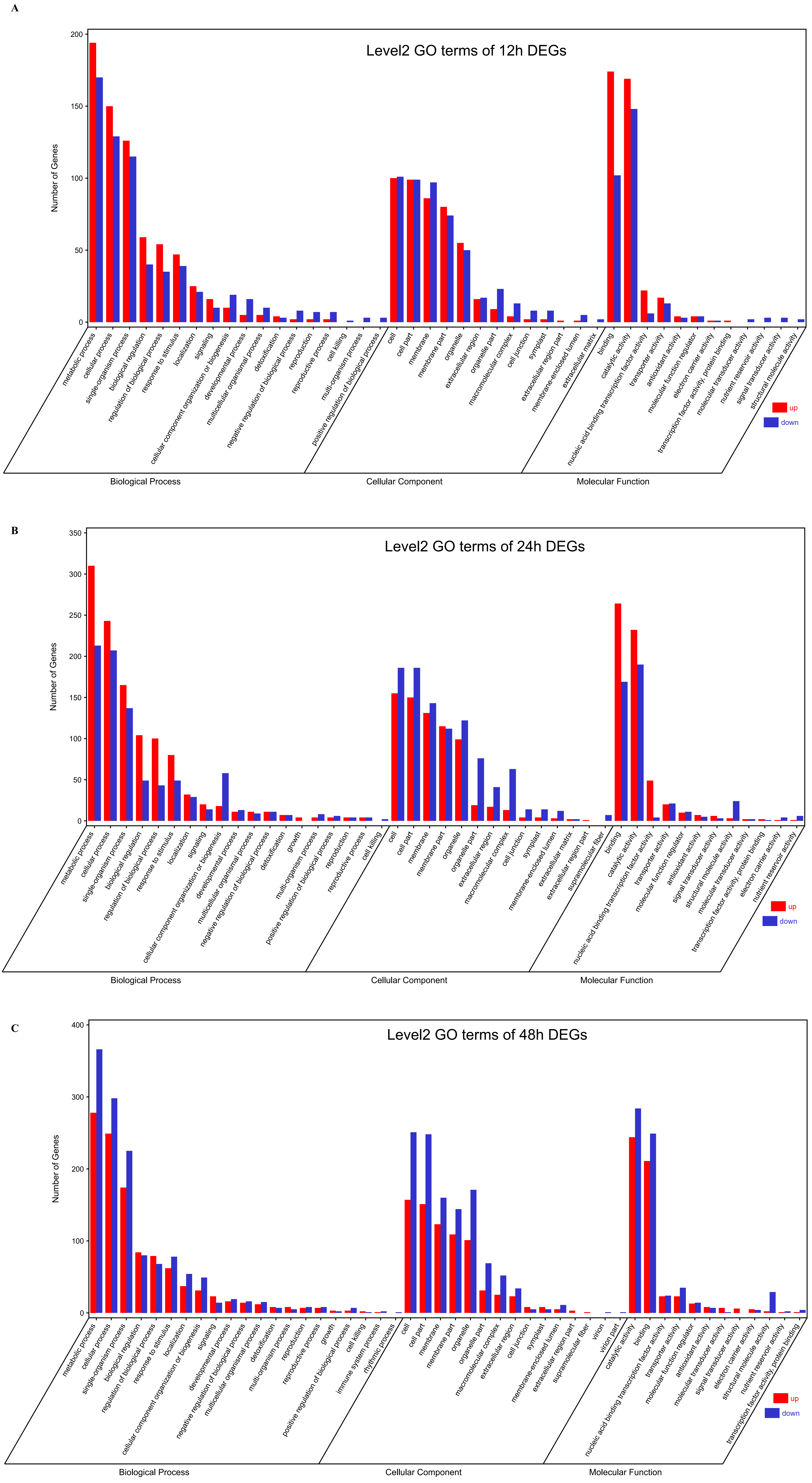

Figure S1. Level 2 GO term categorization of DEGs at 12 h (A), 24 h (B), and 48 h (C). The red bar is up-regulated number of DEGs, and the blue is down-regulated.
