# Supplementary material for: Exogenous 1′,4′-trans-Diol-ABA Induces Stress Tolerance by Affecting the Level of Gene Expression in Tobacco (Nicotiana tabacum L.)
Source: Int J Mol Sci. 2021 Mar 4;22(5):2555. doi: 10.3390/ijms22052555 (PMC7961390; doi:10.3390/ijms22052555)
Supplement: Supplementary file 1 [file ijms-22-02555-s001.zip › ijms-1085705-supplementary/supplementary file 1.docx]

**Supplementary File 1.** **Supplementary Materials and Methods**

**(1) The HR-ESI-MS report about 1’,4’-*trans*-diol-ABA**


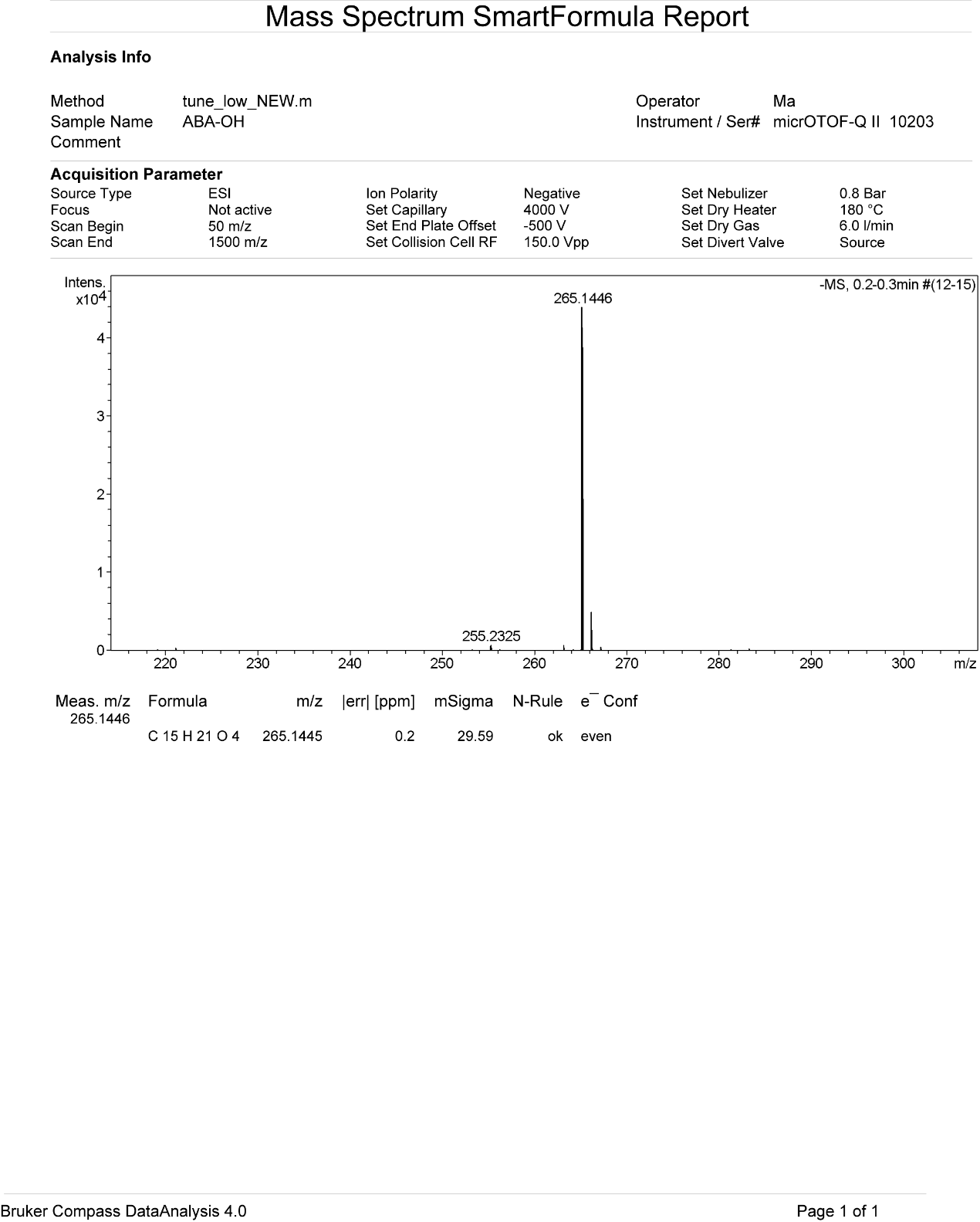


**(2) The information about ^1^H-NMR spectrum (a) and ^13^C-HMR (b) spectrum of 1’,4’-*trans*-diol-ABA**

**a.** ^1^H-NMR(MeOD-d4) δ_H_(ppm): 7.68(1H, d, J=18 Hz, H-4), 6.22(1H, d, J=18 Hz, H-5), 5.70(1H, s, H-2), 5.53(1H, s, H-3′), 4.18(1H, m, H-4′), 2.02(3H, d, H-6), 1.72(1H, H-5′α), 1.65(1H, H-5’β), 1.60(3H, t, H-7′), 1.01(3H, s, H-8’), 0.90(3H, s, H-8′);

**b.** ^13^C-NMR(CDCl_3_) δ_C_(10^6^): 168.34(C- 4’)，150.62(C-1)，140.07(C-2’) , 138.18 (C-3), 127.40(C-5), 126.62(C-4), 116.97 (C-3’), 78.93 (C-2), 65.00(C-1’), 43.54(C-5’), 39.55(C- 6’), 24.21 (C-9’), 21.79(C-8’), 19.98 (C-6), 16.83(C-7’)．
