# Supplementary figures and images for: Exogenous 1′,4′-trans-Diol-ABA Induces Stress Tolerance by Affecting the Level of Gene Expression in Tobacco (Nicotiana tabacum L.)
Source: Int J Mol Sci. 2021 Mar 4;22(5):2555. doi: 10.3390/ijms22052555 (PMC7961390; doi:10.3390/ijms22052555)

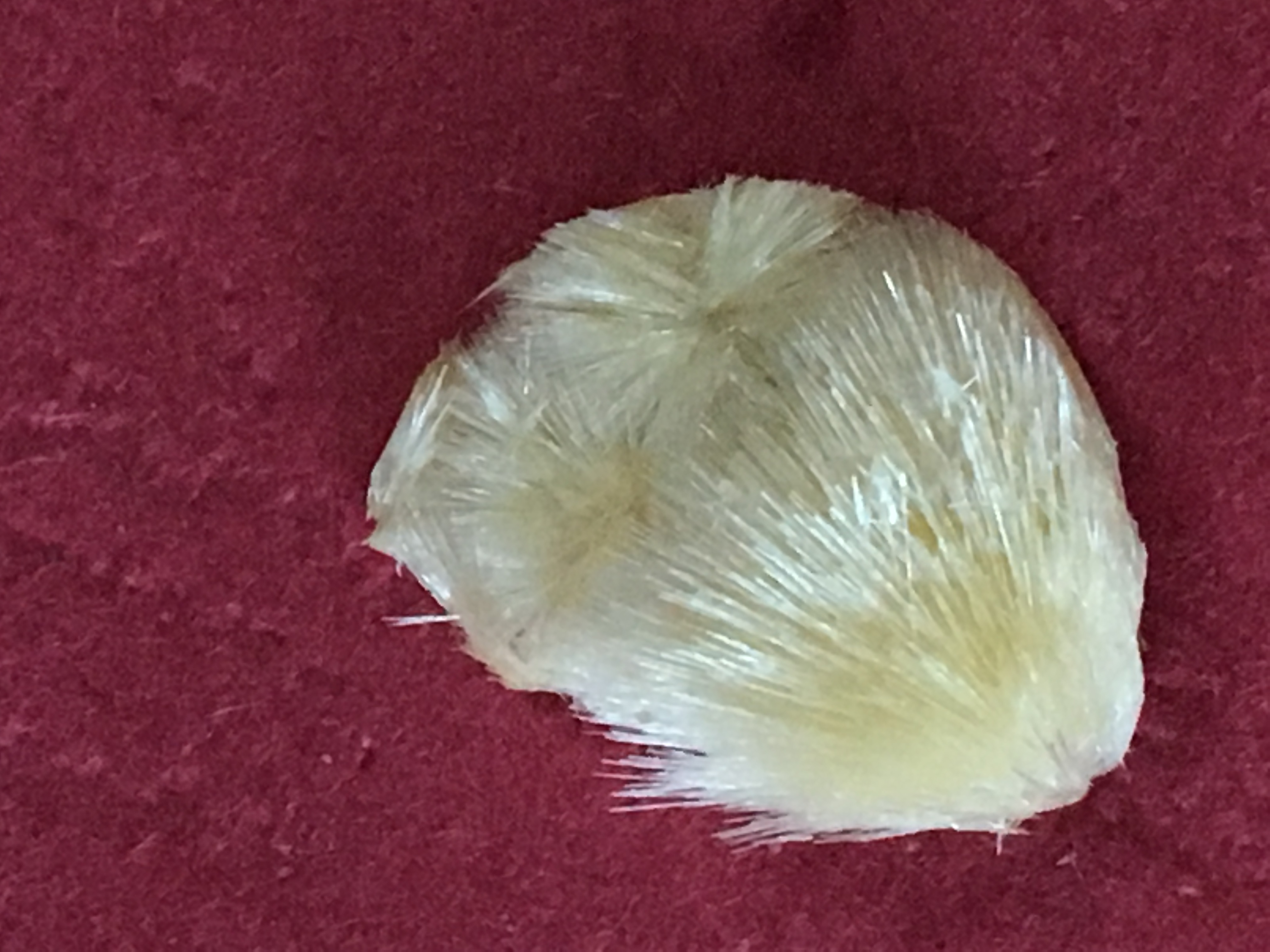

Supplement: Supplementary file 1 [file ijms-22-02555-s001.zip › ijms-1085705-supplementary/Figure S2.JPG]
